# EZH2在肺癌发生发展中的研究进展

**DOI:** 10.3779/j.issn.1009-3419.2016.02.07

**Published:** 2016-02-20

**Authors:** 晖 夏, 长海 于, 文 张, 宝石 张, 英男 赵

**Affiliations:** 100048 北京，解放军总医院第一附属医院胸心外科 Department of Thoracic-cardio Surgery, the First Affiliated Hospital of General Hospital of PLA, 100048 Beijing, China

**Keywords:** 肺肿瘤, 基因治疗, EZH2, Lung neoplasms, Gene therapy, EZH2

## Abstract

肺癌是最常见的恶性肿瘤之一，严重威胁人类生命和健康。筛选合适的靶点是肺癌基因治疗的首要因素。研究EZH2与肺癌发生、发展的分子机制，阐明其信号调控网络，探寻新的治疗靶点和策略，将为筛选临床治疗新靶点提供重要理论支持和实验依据，可以提高肺癌患者生存率、改善生存质量，具有重要的临床应用价值和深远的社会意义。

## Abstract (en)

Lung cancer is a serious threat to human health malignancies upward trend in morbidity and mortality. It is hot topic to investigate the molecular mechanisms of lung cancer development and explore the new therapeutic targets. The underlying mechanism of EZH2 on lung cancer development will demonstrate the new pathway of lung cancer development, invasion and metastasis. The exploration and application of new targeted molecular will improve the survival rate and living quality of lung cancer patients in future.

肺癌是最常见恶性肿瘤之一，严重威胁人类生命和健康，全世界每年新发肺癌病例达120万，患者复发率高、易出现耐药，存活率低，5年生存率仅为20%-30%。肺癌主要分为小细胞肺癌（small cell lung cancer, SCLC）和非小细胞肺癌（non-small cell lung cancer, NSCLC）两个亚型，NSCLC约占80%^[1, 2]^。手术治疗联合化疗和放疗是目前临床肺癌治疗的常规方法，但放疗、化疗副作用严重，患者生存质量差，大多数晚期患者常伴有全身多器官转移，无法进行手术治疗^[3, 4]^。研究肺癌发生、发展的分子机制，探寻新的治疗靶点和策略，提高肺癌患者生存率、改善生存质量，具有重要的临床应用价值和深远的社会意义。

## 1 EZH2与肺癌的进程密切相关

国内外最新研究结果^[5, 6]^表明：EZH2与肺癌发生、发展密切相关，并能预测患者预后水平。研究EZH2在肺癌发生、发展过程中的信号网络和调控机制，是目前国内外研究的热点问题。

### 1.1 EZH2基本结构和功能

人类*EZH2*基因（基因ID：2146），也称ENX-1，定位于人类染色体7q35-7q36区间上。*EZH2*基因组结构中共覆盖76, 939 bp，包括20个外显子和19个内含子。转录后有两种剪接体：转录变异体1，mRNA长2, 695 bp，编码蛋白异构体a含751个氨基酸；转录变异体2，mRNA长2, 563 bp，编码蛋白异构体b含707个氨基酸，与前者相比较，缺少两个结构内片段，编码蛋白异构体略小。EZH2与DNA甲基转移酶（DNA methyltransferases, DNMT）发生相互作用，参与组蛋白去乙酰化酶（histone deacetylase, HDAC）过程，可以影响组蛋白H3K27甲基转移酶的活性^[7, 8]^。

EZH2是果蝇*zeste*基因增强子的人类同源基因，是PcG（polycomb group）基因家族重要成员之一，在PcG基因家族中，起着核心作用。PcG蛋白作为转录抑制因子直接调控涉及分化、发育、细胞命运和干细胞自我更新的基因表达，参与X染色体失活，在肿瘤发生中起着关键调控作用。PcG基因是一进化高度保守基因，包括PRC1和PRC2两种多聚复合物，在生物胚胎发育过程中，通过形成巨大的多蛋白复合物，作用于染色体不同位点，致使染色体变构，从而调控基因表达，维持基因抑制和起始基因的沉默。EZH2分子是PRC2催化活性部分，存在于调节染色质相关基因表达的修饰因子中，在进化过程中具有高度的保守性^[9-12]^。

EZH2的功能与PcG核小体甲基化和DNA甲基化关系密切。研究表明，EZH2可通过甲基化修饰核小体组蛋白H3的27位赖氨酸，丝氨酸/苏氨酸蛋白激酶在第21位磷酸化EZH2，降低PRC2组蛋白甲基转移酶活性，沉默下游相关抑癌基因，如*E-cadherin*、*P16 INK4α*、*P57*、*PSP94*等，促进了肿瘤的发生和发展进程^[13, 14]^。

### 1.2 EZH2表达水平与肿瘤的关系密切

研究发现，EZH2在多种肿瘤组织中高表达，具有促进细胞增殖、加速肿瘤细胞扩散的特点。*EZH2*基因在良性前列腺肿瘤、局限性前列腺癌无表达或弱表达，而在转移性前列腺癌中高表达，与前列腺癌侵袭转移密切相关。正常淋巴器官、淋巴组织的B细胞分化、增殖中，EZH2发挥着重要的作用，与急性髓性白血病、骨髓增生异常综合症、霍奇金淋巴瘤等造血系统肿瘤的发生密切相关，在这些肿瘤中EZH2表达明显增高。据报道，乳腺癌、膀胱癌和大肠癌等肿瘤组织中EZH2高表达，其表达水平与肿瘤直径、分化程度、浸润转移及临床分期等密切相关，与患者预后状况也有关，EZH2高表达者临床预后较差。胃癌肿瘤大小、浸润深度、血管浸润、淋巴结转移、远处转移及临床分期等特征均与EZH2的表达水平高度一致，同时EZH2与ki-67、P53的表达也相关^[11, 12]^。

## 2 EZH2信号通路研究进展

肝脏生理和病理进程中，Wnt/β-catein信号通路扮演着重要的角色，可以调控细胞分化、增殖等过程。据报道，EZH2功能异常影响肝脏肿瘤状态，EZH2可以通过DACT3和DKK1通路，调控Wnt/β-catein表达，影响肿瘤细胞增殖、凋亡等生物过程^[15]^。细胞增殖、生长等生物学功能与PI3K/AKT信号通路密切相关，EZH2的21位色氨酸磷酸化受AKT的调节，影响组蛋白亲和力，与Ras和核因子қB（nuclear factor қB, NF-қB）等信号网络的调控相关，EZH2影响DAB2IP表达，调控下游信号分子，改变细胞增殖与转化粘附功能；EZH2可以影响ADRB2/β-adrenergic通路的功能，调节细胞转化粘附作用。EZH2调控周期蛋白依赖性激酶的磷酸化水平，影响苏氨酸磷酸化^[16]^。

ZH2与相关分子的甲基化水平密切相关。研究发现，EZH2可以影响p16甲基化水平，调节CDK4分子表达，改变细胞增殖的进程。*RAD51*、*FOXC1*和*CDKN*等基因启动子区域H3K27甲基化与EZH2表达水平相关，EZH2通过调节p21表达，调控细胞周期，影响Raf/ERK信号通路，改变细胞生物学特性。此外，EZH2可以调控BMP和NOTCH-1信号分子的作用，影响细胞分化的生物学进程^[16, 17]^。

## 3 EZH2调控肺癌生物学行为的信号通路研究

### 3.1 EZH2在临床肺癌组织中高表达

临床研究结果证实，360例临床Ⅲ期和Ⅳ期肺癌患者中，204例患者EZH2表达水平高于其他患者，患者术后肺癌临床组织标本中EZH2分子高表达，但肺部正常组织中EZH2分子表达为阴性；*Kaplan-Meier*生存曲线分析结果提示，这部分患者生存时间低于EZH2表达水平低的对照组患者，表明EZH2可作为肺癌临床诊断和治疗的靶分子之一。

此外，研究^[18]^还发现EZH2分子表达水平与肺癌患者对化疗药物的耐受性程度密切相关。对顺铂耐药的肺癌患者中，EZH2分子高表达，但其作用机理仍不清楚。

### 3.2 EZH2对VEGF相关信号通路的影响

EZH2在NSCLC中高表达，但其具体作用的分子机制和针对临床治疗的反应特性，目前仍不清楚。据报道，血管内皮生长因子（vascular endothelial growth factor, VEGF）/血管内皮细胞生长因子受体2（vascular endothelial growth factor receptor 2, VEGFR2）信号通路可通过影响E2F、miR-101、缺氧诱导因子-1α等分子，调节EZH2表达。VEGF促进缺氧诱导因子-1α活性，可增加肺癌中EZH2表达水平，降低肺癌中VEGF水平，能够抑制EZH2表达，影响肺癌的生物学进程。降低EZH2在肺癌中表达，能降低体外肺癌细胞的生长状态，这一生物学行为与E2F/Rb信号通路的调控密切相关。Rb是E2F的转录抑制因子，E2F家族的不同亚型E2F1、E2F2、E2F3表达受抑制时，能降低EZH2水平，提示EZH2为E2F下游的信号靶分子。研究^[19-21]^发现，增加转录因子E2F水平可直接提高肺癌中EZH2表达，下调miR-101水平可增加EZH2在肺癌中的表达。

### 3.3 EZH2对细胞周期通路的影响

细胞凋亡标志分子半胱氨酸天冬氨酸蛋白酶-3，肿瘤转移抑制的标志分子DIAB2IP的功能，均与EZH2的过表达密切相关，应用基因沉默的方式降低EZH2表达，可增加半胱氨酸天冬氨酸蛋白酶-3水平，影响肿瘤细胞凋亡，此外还能增加DIAB2IP水平，抑制肿瘤转移进程的发生与发展，而这一功能的变化与ras信号通路密切相关^[21, 22]^。

EZH2参与调控细胞周期调控因子CDK4/6-cyclin D和CDK2-cyclin E的表达，下调EZH2表达可降低肺癌细胞的增殖程度^[21, 22]^。

研究^[23-25]^发现，肺癌组织和肺癌细胞系中均高表达EZH2分子；抑制EZH2表达可增加肺癌放射治疗敏感性，影响p53和p21信号通路，降低细胞周期蛋白Cdc2和Cyclin B1表达，调控细胞周期变化。选择特异性药理学抑制剂或基因沉默的方法，抑制或降低肺癌细胞中EZH2表达水平，能增加肺癌细胞系对顺铂和卡铂的敏感性，诱导细胞凋亡，降低肿瘤细胞增殖程度。EZH2表达与PI3K/AKT信号通路的调控密切相关，可以影响肺癌细胞增殖状态和细胞周期变化，降低动物肿瘤组织体积，延长荷瘤动物生存时间^[19-21]^。以上结果均表明EZH2在肺癌的生物学进程中发挥着关键的调控作用，是肺癌基因临床治疗的可靠靶点之一。

### 3.4 EZH2与miRNA分子的相互作用

研究证明，miR-138分子在SCLC中低表达，与EZH2表达水平密切相关，增加肺癌细胞中miR-138水平，可降低EZH2表达，影响肺癌生物学功能，诱导肺癌细胞凋亡，细胞周期阻滞。动物实验^[23]^表明，增加miR-138，降低EZH2可显著抑制肿瘤体积，提高动物存活率。体内外研究结果均提示miR-138直接参与了EZH2分子调控肺癌发生、发展进程的信号网络，EZH2分子是miR-138的下游直接作用靶点。

最新研究结果^[26]^表明，降低miR-21水平可显著增加肺癌细胞对放射治疗的敏感性，抑制肿瘤细胞增殖水平，增加细胞凋亡，这一过程与PI3K/Akt的抑制密切相关。以上结果提示，EZH2与miR-21之间存在一定联系，可能参与了肺癌生物学进程的信号调控网络，为进一步研究提供了实验基础和方向。

## 4 小结

### 4.1 肺癌的基因治疗

肺癌的基因治疗是一种针对肿瘤细胞相关基因进行特异治疗的新方法、新手段。主要通过基因转导技术应用适宜的载体如腺病毒、脂质体或高分子纳米材料等，将目的基因导入肺癌靶细胞，改变肺癌细胞中相关致癌或抑癌基因的表达，激活或抑制下游信号转导通路，最终达到抑制肺癌细胞生长的目的。如何发现和选择合适的靶基因是肺癌基因治疗的首要因素，是目前国内外研究的热点。

### 4.2 EZH2在肺癌基因治疗中的应用

研究EZH2与肺癌发生、发展的分子机制，阐明肺癌侵袭转移的途径，探寻新的治疗靶点和策略，将为筛选临床治疗新靶点提供重要的理论支持和实验依据，提高肺癌患者生存率、改善生存质量，具有重要的临床应用价值和深远的社会意义。
